# An Unusual Cause of Right Lower Quadrant Pain: The Caecum Diverticulitis

**DOI:** 10.1155/2012/789397

**Published:** 2012-02-13

**Authors:** Murat Yildar, Omer Faruk Ozkan, Kasım Caglayan, Faruk Ozkul, Faruk Cavdar, İsmail Saygın

**Affiliations:** ^1^Department of General Surgery, Medical School, University of Balıkesir, 10145 Balıkesir, Turkey; ^2^Department of General Surgery, Trabzon Numune Training and Research Hospital, 61040 Trabzon, Turkey; ^3^Department of General Surgery, Medical School, University of Bozok, 66900 Yozgat, Turkey; ^4^Department of General Surgery, Mengucek Gazi Training and Research Hospital, Erzincan University, 24100 Erzincan, Turkey; ^5^Pathology Department, Mus State Hospital, 49000 Mus, Turkey

## Abstract

*Purpose*. In the study presented, preoperative examinations and surgical methods were discussed along with literature, regarding two cases who were operated with the prediagnosis of acute appendicitis and for whom caecum diverticulitis was determined. *Case 1*. 21 years old male patient who had applied to hospital with complaint of abdominal pain, underwent an operation with a prediagnosis of acute appendicitis. Right hemicolectomy was performed with mass perioperatively determined in caecum. Histopathological examination revealed necrosis and inflammation in diverticulum wall. *Case 2*. 36 years old female patient applied to emergency department with abdominal pain and underwent an operation with a prediagnosis of acute appendicitis. Appendectomy and diverticulectomy were performed for whom inflame diverticula in caecum was determined perioperatively. Histopathological examination was revealed acute inflammation in diverticulum wall. *Conclusion*. Although solitary caecum diverticulitis is a rarely encountered disease, it must be considered in the differential diagnosis of right lower abdomen pain.

## 1. Introduction

Solitary caecum diverticulitis which was described primarily by Potier in 1912 is more frequently encountered in Asian societies than Western societies [1]. Although etiology of caecum diverticulitis is not clarified completely, it is generally regarded as congenital and it includes all the layers of colon wall [2, 3]. Because symptoms and clinical findings of caecum diverticulitis show similarity with acute appendicitis its diagnosis before surgery is difficult and therefore its actual prevalence is not known. However, in the cases that were operated with diagnosis of acute appendicitis, caecal diverticulitis was determined in the ratio of 1/300 [4]. Optimal treatment of caecum diverticulitis is disputable. While some authors accept surgical treatment due to its high relapse and complication rates; some state that medical treatment is active and safe due to its low recurrence rates [3, 5].

Diagnosis processes and treatment methods of two cases that were operated with acute appendicitis and where caecum diverticulitis was determined during operation were presented together with literature in this study.

## 2. Case 1

**Figure 1:**
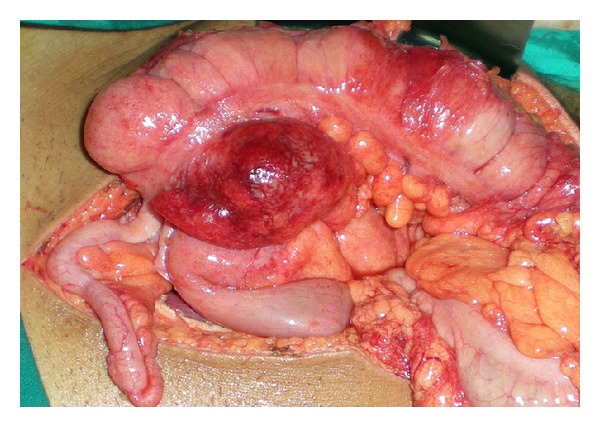
Intraoperative image of inflamed cecal mass formed by solitary caecal diverticulitis.

**Figure 2:**
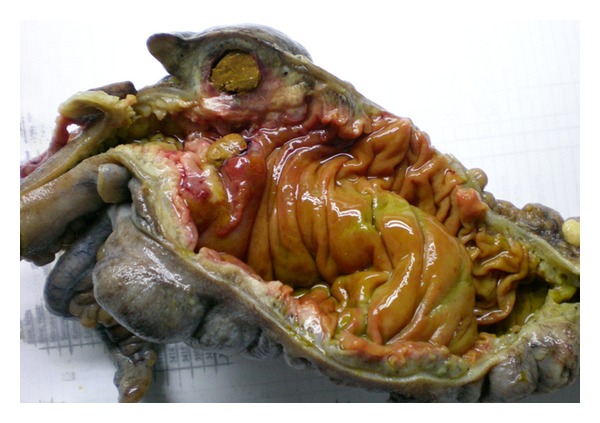
Macroscopic image of resection piece with diverticulum in caecum, thickening in diverticulum wall, and fecaloma within.

**Figure 3:**
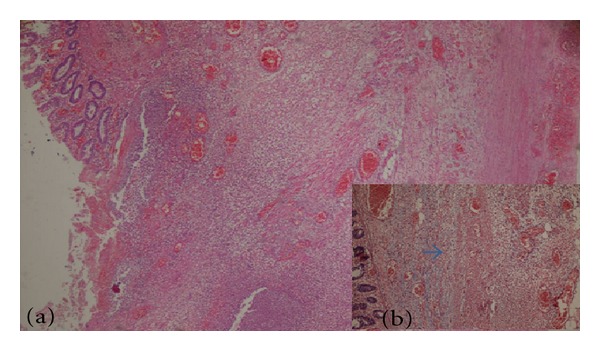
Histopathological images of resection piece (focal mural infarct findings, congested vascular structures along complete intestine wall, muscularis propria that shows continuity along intestine line on which diverticulitis formed and that is made clear with histochemical Masson-Trichrome, also shown in (b), oedema and inflammation findings attracted attention.). (a) focal mural infarct (HE X 40), (b) muscularis propria, indicated with blue arrow (Masson-Trichromex 100).

21 years old male patient applied to emergency department with complaints of abdominal pain, nausea, and vomit that started 2 days ago. There was no specialty in the personal and family history of the patient. Blood pressure, pulse, and axillary temperature were, respectively, determined as 120/80 mm Hg, 96/minute, and 38.2°C. Sensitization, defense, and rebound findings were determined in the right lower quadrant during abdominal examination. In the laboratory examination of the patient, biochemical and complete urine analysis were in normal limits and leucocyte count was 12100 K/uL (normal range: 4600–10200). In the radiological examination of the patient, the erect abdominal radiography revealed no specialty. In the abdominal ultrasonography (USG), free fluid between intestinal loops in the right lower quadrant and mesenchymal lymphadenopathy was determined. The patient was taken into operation with pre-diagnosis of acute appendicitis and abdominal cavity was entered with Mc Burney incision. 15–20 cc fluid in serous quality was detected in appendix lodge in exploration. Appearance of appendix was normal. 7 cm long and inflame mass was determined in the proximal of ileocecal valve in the continuation of exploration (Figure 1). In addition, subumbilical median incision was made to the patient due to these findings. Since benign/malign distinction of the lesion could not be done, right hemicolectomy and ileotransversostomy were performed to the patient. The patient was discharged from the hospital on the postoperative 7th day without problems. Fecaloma-related diverticulitis was monitored in diverticulum in caecum in the macroscopic examination of the piece (Figure 2) and intense inflammation and necrosis were monitored in the wall of diverticulum in the histopathological examination of the piece (Figure 3).

## 3. Case 2

Blood pressure, pulse, and axillary fever of 36 years old female patient who had applied to emergency department with the pain that started approximately the day before around belly and localized to right lower quadrant were, respectively, 110/70 mmHg, 102/minute, 38.0°C. Sensitization, defense, and rebound findings were determined in the right lower quadrant during abdominal examination. In the laboratory examination, biochemical, and complete urine analysis were in normal limits and leucocyte count was 17000 K/uL (normal range: 4600–10200).

In the radiological examination of the patient, the erect abdominal radiography revealed no specialty. In the USG performed, free fluid between intestinal loops in the right lower quadrant was determined. The patient was taken into operation with prediagnosis of acute appendicitis and abdomen was entered with Mc Burney incision. Retrocecal localized appendix was monitored normal and pericecal minimal free fluid in serous quality was seen in exploration. Inflame caecum diverticulitis with a root of 1 cm diameter, 1,5 cm long and 1 cm diameter was determined in the continuation of exploration, on the front wall of caecum, 1 cm proximal to ileocecal valve, under inflame epiploic appendix. Appendectomy and diverticulectomy were performed. The patient, for whom oral food was started in 12 hours, was discharged from hospital on the 2nd day with complete surgical healing. In the histopathological examination of the resection piece, findings of distinct acute inflammation were determined on the diverticular wall.

## 4. Discussion

Approximately 80% of caecum diverticulitises are anatomically 1-2 cm away from ileocecal valve and approximately 60% of them are seen on the front side of caecum [2]. In case inflammation of diverticula localized on the front side of caecum occurs, perforated and generalized peritonitis table forms; posterior localized cases may rather imitate clinically perforated colon carcinoma as a mass [2]. Preoperative diagnosis of caecum diverticulitis is difficult because symptoms show similarity with acute appendicitis. Most of the authors state that to make a distinction between acute appendicitis and caecum diverticulitis preoperatively is very difficult due to the similarity between symptoms, but some authors mention that longer duration of disease than appendicitis, no nausea and vomiting, less toxic characteristics are the distinctive features of diverticulitis. However, to distinguish between these two entities is very difficult and there is not any clinical finding or diagnosis test to diagnose caecum diverticulitis precisely. Despite clinical, laboratory, and all radiological examinations, more than 70% of these cases were operated due to acute appendicitis [4, 5]. Only 9% of caecum diverticulitis cases are diagnosed accurately before operation and appendectomy are performed to most of these cases [2].

Right colonic diverticulitis may be diagnosed preoperatively with colonoscopy and contrast enhanced colonography. However, in the case of diverticulitis due to the probability of perforation or protruding of barium out of lumen, these examinations are contraindicated at emergency [6]. In the preoperative correct diagnosis of caecum diverticulitis, USG and contrast enhanced computerized tomography are useful [4–6]. USG may give direct or indirect information about acute caecum diverticulitis. Circular or elliptic hypoechoic or anechoic area on the wall of colon that is thickened segmentally is an important sonographic finding [7]. Chou et al. [7] reported that they could distinguish between acute appendicitis and right colon diverticulitis with 100% accuracy rate with abdominal USG in 934 patients applied with pain of right lower quadrant. But this study, stating that USG can be used with 91.3% precision and 99.8% selectivity in the diagnosis of caecum diverticulitis, is not confirmed with other studies [8]. This difference may be due to the experience of person performing ultrasound. Hence, although USG was performed to both cases presented in our study, any finding related to diverticulitis was not determined.

Jang et al. stated in their study that differentiation between diverticulitis and carcinoma could be made with thin slice CT with 92.5% accuracy rate [9]. And in another study they stated that CT was 85% precise, 68% selective, 28% positive predictive, 97% negative predictive, and 70% diagnostically accurate for right colon diverticulitis [8]. Thickening in intestine wall at right colon level, pericolonic fat infiltration, pericolonic abscess, and extraliminal air are the findings of right colon diverticulitis in CT. However, these are nonspecific and may be seen in cancers of ileocecal area [6].

Because there is acute abdominal pain in most of the cases with caecum diverticulitis, operation decision is taken based on clinical examination and laboratory findings without employing imaging methods [10]. When literature regarding treatment of caecum diverticulitis was investigated, it is seen that there is a wide spectrum from conservative medical treatment to right hemicolectomy [1–6, 10, 11]. Although there is no consensus for the treatment of caecum diverticulitis, conservative treatment is advised generally for the cases for which preoperation diagnosis is established and which are not complicated, and surgical treatment is advised for the cases complications like perforation and abscess forms [3, 12, 13]. Surgical treatment alternatives like diverticulectomy, ileocolic resection, or right hemicolectomy are stated but the surgical method performed must be determined based on the peroperative findings [14]. Yang et al. [12] stated in their study that in case perioperative malignancy was suspected, it indicated colectomy, Fang et al. [5] advised right hemicolectomy as definitive treatment, Papaziogas et al.[10] stated that diverticulectomy was sufficient. In the study presented right hemicolectomy was performed to one of the cases because a mass was detected during operation and carcinoma suspect could not be excluded; the other case was treated with diverticulectomy, offered in literature which was a limited surgical method together appendectomy procedure because there was not any carcinoma doubt.

In conclusion, when caecum diverticulitis is considered to be more frequently seen in Asian societies, it must be taken into account in differential diagnosis especially in patients with appendectomy, atypical symptoms, and right lower quadrant pain associated with acute appendicitis. Since diagnosis before operation will completely change the treatment method differential diagnosis must be established with radiological methods in these cases. If diagnosis is established during operation when there is no doubt about perforation, mass formation, or carcinoma, limited resection like diverticulectomy must be performed.
